# Expanded Role of a Dietitian in Monitoring a Gluten-Free Diet in Patients with Celiac Disease: Implications for Clinical Practice

**DOI:** 10.3390/nu13061859

**Published:** 2021-05-29

**Authors:** Katarzyna Gładyś, Jolanta Dardzińska, Marek Guzek, Krystian Adrych, Zdzisław Kochan, Sylwia Małgorzewicz

**Affiliations:** 1 Department of Clinical Nutrition, Medical University of Gdansk, 80-211 Gdańsk, Poland; annadar@gumed.edu.pl (J.D.); zdzislaw.kochan@gumed.edu.pl (Z.K.); sylwia.malgorzewicz@gumed.edu.pl (S.M.); 2 Department of Gastroenterology and Hepatology, Medical University of Gdansk, 80-211 Gdańsk, Poland; marek.guzek@gumed.edu.pl (M.G.); krystian.adrych@gumed.edu.pl (K.A.)

**Keywords:** celiac disease, gluten-free diet adherence, dietary assessment, dietary reference intake

## Abstract

Access to a registered dietitian experienced in celiac disease (CD) is still limited, and consultation when available focuses primarily on the elimination of gluten from the diet. Thus, the aim of this study was to evaluate the nutritional value of a gluten-free diet (GFD) in adult CD patients before, and one year after, the standard dietary education. The study included 72 CD patients on a GFD and 30 healthy controls. The dietary intake of both groups was assessed through a 3-day food diary, while adherence to a GFD in celiac subjects was assessed using Standardized Dietician Evaluation (SDE). Subsequently, all CD patients received detailed education on gluten sources, and 48 of them participated in a one-year follow-up. Results: Comparison with the control group showed that consumption of plant protein in CD patients was significantly lower, whereas fat and calories were higher. At baseline, only 62% of CD patients adhered to a GFD, but the standard dietary education successfully improved it. However, the nutritional value of a GFD after one year did not change, except for a reduced sodium intake. The CD subjects still did not consume enough calcium, iron, vitamin D, folic acid or fiber. In conclusion, while the standard dietary education improved GFD adherence, it did not significantly alter its nutritional value. Therefore, it is necessary to increase the role of a dietitian in the treatment of CD.

## 1. Introduction

Celiac disease (CD) is an autoimmune disease that affects the small intestine in genetically predisposed people after consuming gluten [1] and occurs in about 1% of people in most populations [1]. Although new therapeutic strategies (gluten proteolysis, intestinal tissue transglutaminase 2 inhibitors, probiotics, immunotherapeutic methods) have been tested, the primary treatment for CD is still a gluten-free diet (GFD) [2]. It leads to resolution of symptoms, intestinal mucosa recovery and increased absorption of nutrients. The diet of a CD patient has to be based on grains such as maize, buckwheat, millet, rice, amaranth, tapioca or teff; however, gluten-containing whole grains generally have higher amounts of fiber and nutrients such as B vitamins, calcium and iron, so how to balance a GFD must be addressed [3] because nutritional deficiencies may increase the risk of many CD complications: osteoporosis and osteopenia, micro- and macrocytic anemia, chronic weakness or neurological symptoms, such as peripheral neuropathy and numbness [4]. On the other hand, replacement of whole grain barley, rye and wheat products with gluten-free equivalents may be associated with the increased consumption of fats, especially saturated fatty acids (SFA) and trans fats, as well as salt, sucrose and phosphorus. This can lead to the development of metabolic disorders like obesity, dyslipidemia, gout, diabetes, hypertension and other cardiovascular complications [5]. An extensive fat intake can also reduce the absorption of other nutrients like magnesium [6]. Furthermore, high fat consumption together with low fiber ingestion led some CD patients, who adhered strictly to a GFD, to develop persistent symptoms such as bloating or abnormal bowel movements [7,8]. For all of the aforementioned reasons, it seems warranted that the dietary control of CD should be more detailed. The standard dietary education of patients with celiac disease usually focuses only on the proper recognition and avoidance of gluten sources; consequently, some patients can choose food that is gluten-free, but highly processed with low nutritional value. Unfortunately, there is very little research on the nutritional value of GFDs in adults. It is also unclear if the dietary education focusing on the elimination of gluten sources affects the nutritional value of this diet. Therefore the aim of the present study was to assess the nutritional value of a GFD in adult patients before and one year after standard dietary education.

## 2. Materials and Methods

### 2.1. Study Population

The study was conducted from October 2015 to April 2018 and involved 72 adults (63 women and 9 men) with a diagnosis of CD who were outpatients under the care of the Department of Gastroenterology and Hepatology at the Medical University of Gdansk. They were randomly recruited by a gastroenterologist during a routine consultation and had to meet the following inclusion criteria: be on a GFD, be over 18 years of age and have a diagnosis of CD based on serological and histological tests according to the British Society of Gastroenterology guidelines [9]. The exclusion criteria were being under 18 years, not giving consent to take part in the study, being pregnancy and not having a clear diagnosis of CD. Based on the patients’ anamneses, the other most common autoimmune disease was hypothyroidism (15% of participants). A positive result for anti-endomysial antibodies (EMA) and anti-tissue transglutaminase (tTG) IgA together with a biopsy result were adopted as indicators of active disease. In the end, the study group consisted of 26 subjects with active CD and 46 with CD in remission.

The control group consisted of 30 gluten-eating healthy adults matched by age and sex with negative results for tTG-IgA antibodies. All controls were randomly recruited based on an online advertisement posted on the university’s social network. They followed a traditional diet without any food elimination or therapeutic modifications. Written consent was obtained from all the participants. Pregnant women were excluded. The project was approved by the university bioethics commission (MUG Bioethics Committee approval number is NKBBN/403/201).

### 2.2. Serologic and Histologic Tests

In all CD patients, blood was drawn to determine serum levels for EMA and anti-deamidated gliadin peptide (DGP), and a duodenal biopsy was performed, whereas tTG antibodies were checked for both the controls and studied subjects. DGP and tTG antibodies were measured using the enzyme-linked immunosorbent assay (Euroimmun, Wrocław, Poland), while EMA antibodies were assessed using an indirect immunofluorescence technique (Euroimmun, Wrocław, Poland) in the hospital laboratory. The titers were considered positive according to the manufacturer’s specifications. No CD subjects had IgA deficiency.

### 2.3. Dietary Assessment and Education

Dietary intake was assessed through a 3-day food diary which consisted of an accurate description of food intake during two weekdays and one weekend day. The dietitian informed all participants (studied and controls) by telephone how to prepare the food diary before the face-to-face consultation. During a personal appointment, a registered dietitian experienced in the dietary management of gastrointestinal disorders validated the diary by using 24 h diet recall and asking detailed questions about the use of condiments, cooking methods and brands of foods. The amounts of food were assessed by showing the participants a photographic atlas published by National Food and Nutrition Institute (Poland) [10]. To estimate the intake of energy, macro- and micronutrients, the same dietitian used specialized software (Dietetyk 2012 JuMaR, Warsaw, Poland) based mainly on Polish Food Composition databases published by the National Food and Nutrition Institute in Warsaw, Poland, and on the United States Department of Agriculture database [11,12].

The basal metabolic rate (BMR) was calculated using the Harris–Benedict Equation, as follows:

- For men: BMR = 10 × weight (kg) + 6.25 × height (cm) **−** 5 x age (years) + 5
- For women: BMR = 10 × weight (kg) + 6.25 × height (cm) **−** 5 x age (years) **−** 161

A physical activity level (PAL) was determined for each person to estimate total energy expenditure. The percentage of the dietary reference intake (DRI) for energy and micronutrients was calculated based on the following nutrition standards for the Polish population: (Observed amount/reference amount) × 100 [13]. The following vitamin and mineral figures from the recommended daily allowance (RDA) were used: Vitamin A (as retinol activity equivalents), C, B_1_, B_2_, niacin, B_6_, B_12_, folate (as dietary folate equivalents) and calcium, phosphorus, magnesium, iron and zinc. For nutrients that do not have RDA values (vitamins D and E, sodium, potassium, fiber) adequate intake (AI) was adopted. The intake norms for fats, protein and carbohydrates according to Polish standards are presented as a percentage of the energy derived from them. 

In the CD group, GFD adherence was also evaluated by the standardized dietitian evaluation (SDE), which is considered to be the gold standard for testing compliance with a GFD [14]. The usefulness of applying this method in the Polish population with CD was presented in our previous work [15]. The SDE consists of a 3-day diary, food label quiz and a detailed interview conducted by a trained dietitian about reading medicine labels, dietary supplements, eating out and the risk of gluten cross-contamination. The results were presented on a 6-point Likert scale: 1 point—perfect GFD adherence; 2 points—good GFD adherence; 3 points—fair GFD adherence; 4 points—poor GFD adherence; 5 points—very poor GFD adherence, and 6 points—no GFD. 

**Figure 1:**
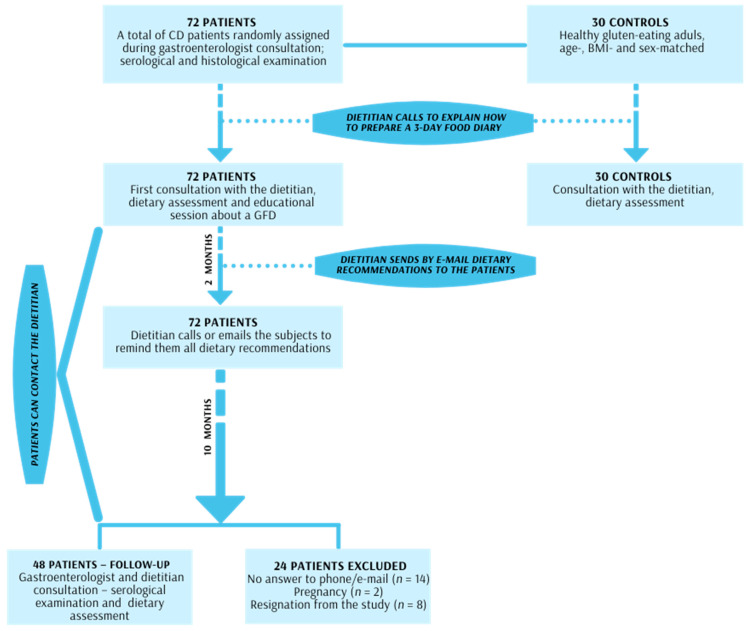
Scheme of the study.

During the consultation, the dietitian also provided an individual one-hour detailed education about the GFD with particular emphasis on hidden sources of gluten. After the meeting, dietary recommendations were sent by e-mail to the patient to summarize the information about gluten sources: a list of manufacturers of gluten-free foods, local stores that specialized in gluten-free products and a list of gluten-free food additives and ingredients. Additionally, two months after the educational consultation, the dietitian called or emailed the CD subjects to remind them of all the dietary recommendations. Each patient could also contact the dietitian for one year if any questions about the GFD diet had arisen. The SDE with a 3-day food diary was repeated one year after the education. The scheme of the study is presented in Figure 1.

### 2.4. Anthropometric Measurements

Anthropometric measurements were collected by the same dietitian during the first and second face-to-face consultations. Body weight was measured using a body composition analyzer (Jawon Medical X-Contact 350, Daejeon, Korea), while height was measured to the nearest 5 mm using a stadiometer (SECA 213, Hamburg, Germany). The body mass Index (BMI) was calculated from weight and height (kg/m^2^) and values were categorized according to World Health Organization criteria.

### 2.5. Data Analyses

The data are expressed as mean ± SD or median and interquartile range (Q1–Q3). The results of the SDE were additionally divided into two groups: good (Good–Perfect) and bad (Fair–Poor–Very Poor). A Kolmogorov–Smirnov test was used to verify whether the variable distribution was normal. The differences between groups were evaluated by an independent Student’s *t*-test and U Mann–Whitney test (when the distribution of the variable was not normal) or chi-square tests, as appropriate. Data before and after dietary education were compared with the use of the paired Student’s *t*-test or Wilcoxon signed-rank test (when the distribution of the variable was not normal). Statistical analysis was performed using STATISTICA version 13.3 (StatSoft, Kraków, Poland), and *p* values < 0.05 were considered statistically significant.

## 3. Results

### 3.1. Characteristics of the CD Patients and Control Subjects

The characteristics of the studied population are presented in Table 1. Although the mean time from the diagnosis of CD was 4.8 ± 7.0 years, only 17 of 72 patients (24%) admitted that they had never been consulted by a dietician prior to the study.

### 3.2. Analysis of Energy and Nutrient Intake in all CD Patients (n = 72) and Control Group (n = 30)

The intake of energy and nutrients assessed through a 3-day food diary in all CD patients and healthy controls is presented in Table 2 and Table 3.

As can be seen in Table 2 and Table 3, consumption of plant protein in CD patients was significantly lower, whereas consumption of fat and calories was higher than in the control group. There were no other differences between the groups, but the intake of fiber, calcium, iron, vitamin D and folic acid was too low according to the DRI in both CD patients and the control group.

The diet of CD patients who were diagnosed more than one year before the study showed differences in energy consumption from those who had a shorter disease duration. They had, respectively, a higher proportion of energy from fats (39.7% ± 9.1 vs. 34.8% ± 10.4, *p* = 0.04) and MUFAs (14.8% ± 5.8 vs. 10.6% ± 5.0, *p* = 0.006) but lower energy from carbohydrates (44.3% ± 9.7 vs. 50.2% ± 10.2, *p* = 0.04).

### 3.3. Analysis of Energy and Nutrient Intake in Two Subgroups of CD Patients: in Remission (n = 46) or with Active CD (n = 26) in Comparison to Control Group (n = 30)

No differences were observed among the three groups in age, sex or BMI. There were was also no difference in the macro- and micronutrient composition of the diets of patients in remission or with the active CD.

In a comparison of the nutritional value of the diet of patients with active CD to that of the control subjects, a lower proportion of energy from plant protein was found in the active CD group: (25% ± 14 vs. 30% ± 11, *p* = 0.04) and PUFA (4.5% ±2.0 vs. 5.6% ± 1.9, *p* = 0.04). 

It was observed that CD participants in remission consumed more energy than control subjects (2135 ± 764 vs. 1822 kcal ± 451, *p* = 0.047). On the other hand, a lower percentage of energy from plant protein was observed in CD patients in remission than in the control group (24% ± 12 vs. 30% ± 11, *p* = 0.007).

### 3.4. Assessment of Adherence to a GFD in all CD Patients (n = 72) at Baseline

**Figure 2:**
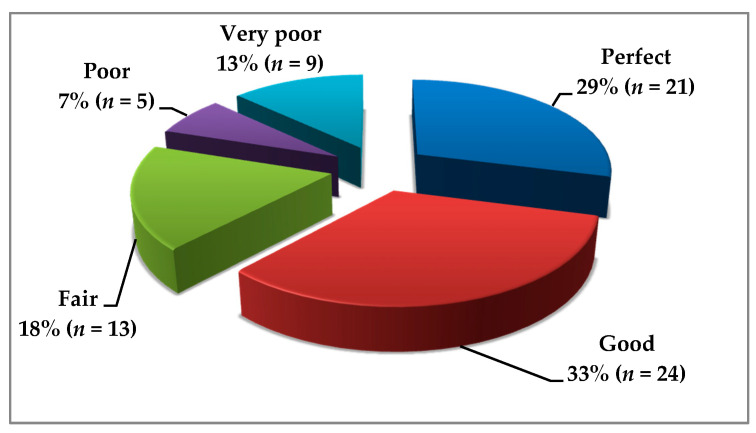
Adherence to a GFD in CD patients (*n* = 72) assessed by the Standardized Dietitian Evaluation (SDE).

The results of the SDE are presented in Figure 2, and showed that 62% (*n* = 72) presented perfect or good adherence to a GFD. The median SDE score was 2 and the interquartile range (Q1–Q3) was 1–3. The SDE score did not differ for those who admitted to having met a dietitian prior to the study to those who had never met one. The results of the SDE also did not differ among those who had a disease duration shorter or longer than one year, but the patients in CD remission more often presented perfect or good adherence to a GFD than those with an active disease (74% vs. 42%, *p* = 0.008). 

### 3.5. Analysis in Follow-Up CD Group (n = 48)

Forty-eight participants of the CD group (44 women, 4 men) gave their permission to attend follow-up consultations with a gastroenterologist and a dietitian after one year to re-evaluate the serology, dietary intake and nutritional status using the same methods as during the first appointment. 

#### 3.5.1. Changes in Nutritional Status, Adherence to a GFD and Autoantibody Levels after One Year of Follow-Up

Patients in the follow-up CD group (*n* = 48) had a higher BMI one year after dietary education (21.8 kg/m^2^ ± 3.3 vs. 22.4 ± 3.3, *p* = 0.001). 

**Figure 3:**
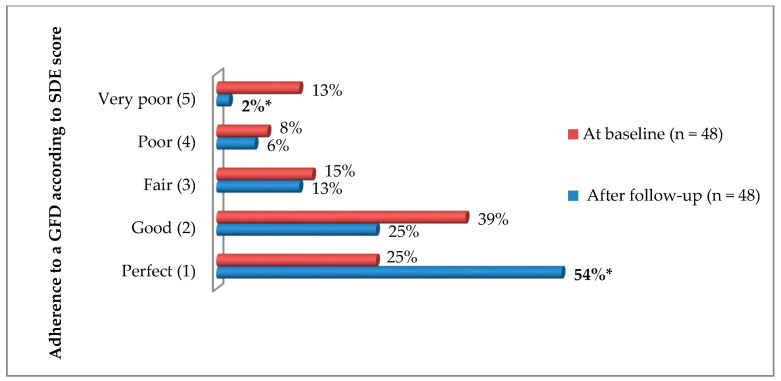
The adherence to a GFD assessed by the Standardized Dietitian Evaluation (SDE) in follow-up CD patients (*n* = 48) before and one year after education. * Differences were statistically significant (*p* < 0.05).

The changes in adherence to a GFD in the whole follow-up group are presented in Figure 3. Based on the SDE results, 60% (29/48) presented better adherence (a lower SDE score) than at baseline, and only 8% (4/48) were given a worse SDE score. In a subgroup of CD patients with a bad SDE score (between 3 and 5, *n* = 17) at the beginning of the study, as many as 53% (9/17) followed a GFD perfectly or well (SDE score 1 or 2) one year after the education. The median SDE score for the whole follow-up group decreased from 2 points to 1 point (*p* = 0.0001). The serum titers of EmA IgA, tTG IgA and DPG IgA also significantly decreased in the follow-up group (*p* values, were, respectively 0.04, 0.02, and 0.0001). 

#### 3.5.2. Energy and Nutrient Intake in Follow-Up CD Group

A comparison of the nutritional value of the GFD in CD patients (*n* = 48) at the beginning and after one year is presented in Table 4 and Table 5. As can be seen, there were no significant differences between the baseline and after follow-up consumption of macro- and micronutrients, except for reduced sodium intake. According to the DRI, the CD subjects still did not consume enough fiber, calcium, iron, vitamin D or folic acid.

## 4. Discussion

It is well known that the main treatment for CD until now has been lifelong compliance with a GFD. Poor dietary adherence can cause serious health problems, such as the risk of T-cell lymphoma and other autoimmune diseases [16]. However, many adults with CD still misidentify gluten sources with adherence rates ranging from 42% to 91% depending on the study method [17]. In the present study, we used the Standardized Dietician Evaluation, which assesses compliance with a GFD very accurately, and found that only 62% of CD patients followed a GFD properly. Hence we confirmed that the lack of adherence is still a problem. Monitoring compliance with a GFD is critical for achieving serological and histological remission of the disease, but it may not be sufficient to improve the long-term prognosis. Some studies even suggested that a GFD may increase the risk of obesity and type 2 diabetes [5,18]. Therefore, in our current study, we compared the nutritional value of the GFD in adult CD patients with the diet of controls. We also wanted to know if the dietary education provided by a registered and highly experienced dietitian who focuses mainly on identifying and avoiding gluten sources would change the nutritional value of the diet in CD patients. Our results showed that while such education did improve adherence to a GFD, it did not significantly alter the nutritional value of the diet. After a year, CD patients consumed less sodium, but still ate too much fat, especially SFA and not enough fiber. It is difficult to compare these results with other studies because of a lack of follow-up studies to assess the nutritional value of a GFD after education from a dietitian. A small number of studies focused on the nutritional aspects of a GFD that included children, for whom access to dietary consultations is often easier.

Our observation that improved adherence to a GFD after a dietitian’s consultation did not always guarantee better nutritional value is quite similar to that of Sepherd et al. [19]. They applied education focused primarily on gluten avoidance in newly diagnosed CD patients, and the nutritional value was checked before starting the GFD and after 12 months. It was found that nutrient intake (except for starch) did not change after the education. The authors also diagnosed many nutritional inadequacies in patients with long-term treated CD concerning, e.g., vitamin A, thiamin, fiber, folate, calcium and iron. Our patients also had inadequate intake of fiber, calcium, iron and folic acid. Based on our results, we agree with others that the extension of standard education to teaching patients about the quality and nutritional value of gluten-free foods is even more important [19,20]. As can be seen from our study, compliance with a GFD is not the only issue for treating CD patients. Those who only follow a GFD but do not eat well-balanced meals might be at risk for metabolic disorders. Our analysis showed that, in general, CD patients consumed more fat and calories and less plant protein compared to the control subjects. This observation was also in line with other studies for probably two reasons [21,22]. First, foods dedicated to CD patients, especially highly processed, typically still contain higher amount of fats and calories than standard gluten-containing foods because substituting gluten often requires the manufacturer to use more ingredients or food additives [23]. Secondly, CD patients often turn to gluten-free snacks rich in fat, calories and sucrose to improve their mood or because they are simply available and readily certified. In our study we observed that the persistent overconsumption of fat and calories in CD patients led to an increase in the BMI. Similarly, in the study of Mahadev et al., nearly half of CD patients gained weight after starting a GFD [24]. On the other hand, the higher BMI value after follow-up may also be the result of improved intestinal absorption due to reduced inflammation of the duodenum.

It should be emphasized that inadequate nutrient intake was also observed in our healthy control group. Both groups consumed on average more than 300 mg of cholesterol per day, which the European Food Safety Authority (EFSA) and Polish Diabetes Association say increases the risk of metabolic disorders such as diabetes, hypertension, atherosclerosis and stroke [25,26]. Similarly, both CD patients and controls consumed more fat than recommended, which may indicate that overconsumption of fat is a problem not only for people with CD, but also for society as a whole [27]. The study conducted by the Polish National Research Institute confirmed that per capita fat intake in Poland increased from 23.6 kg to 33.5 kg from 1990 to 2015 [28]. It has also been observed that a healthy population consumes more products containing plant fats. In our study, we also noticed that the control group consumed more PUFAs than the patients with active CD, but there was no difference in PUFA intake between CD patients in remission and controls. It should be mentioned that patients with the properly treated CD do not need to restrict fat because they are free from gastrointestinal symptoms and fat is gluten free. Additionally, plant fat is very rich in unsaturated fatty acids, which are increasingly popular with people interested in healthy eating, including those on a GFD. This may explain the increased fat intake, especially MUFAs, which was observed in patients with CD lasting more than a year. In contrast, typical carbohydrates are grains, most of them gluten containing, so we found that, over time, CD patients reduced their intake in favor of fats.

Reduced plant protein intake, which was observed in both patients in remission and with active CD, may be due to a decreased consumption of grains for fear of gluten contamination or to buying products labeled both gluten free and PKU (a low-protein diet for patients with phenylketonuria), such as bread. In healthy people, gluten is one of the main plant proteins consumed every day since it is found in wheat or rye bread, pasta, barley groats and various baked goods. In addition, people with CD often rely on gluten-free products made from white rice or corn, which contain less plant protein than wheat. Importantly, a good alternative to corn or rice might be buckwheat, oat, teff, amaranth and quinoa. Despite the fact that high nutritional value has made pseudocereals a modern trend in the human diet, in some countries such as Poland the availability of teff or quinoa is still limited partly due to the high price [29]. The lack of reimbursement for gluten-free foods in Poland makes them even more difficult to purchase. The situation is much better with buckwheat, amaranth and gluten-free oat products, because they are easily available and relatively cheap. It is worth pointing out that the market for gluten-free products has been growing steadily in recent years, and the situation is already more favorable than it was 10 years ago [30].

We also found that the patients with CD did not consume enough fiber, calcium, iron, vitamin D, or folic acid although it was seen that the same problem occurred in the control group. Fiber deficiency can lead to impaired intestinal peristalsis as well as dysbiosis of intestinal microbiota [31]. As for the subsequent nutrient deficiencies (calcium, iron, vitamin D, folic acid), patients with CD should pay special attention to them due to possible malabsorption. These patients are also at risk of osteoporosis and osteopenia, anemia, and neurological disorders [32,33]. Low calcium intake may be the consequence of lactose intolerance, which is more common in celiac disease patients than the general population. On the other hand, we noted that, like Zingone et al., controls also consumed insufficient amounts of calcium [34]. In CD patients who cannot achieve adequate intake via a GFD or with documented low serum levels, calcium and vitamin D should be supplemented [16].

Some of the nutritional deficiencies in GFD may also be the result of discrepancies in food fortification policy [35]. Gluten-free products are not fortified in the standard way as conventional foods, so they may contain less fiber, iron, or B vitamins. Therefore, the task of a dietitian is also to show particularly malnourished patients what gluten-free products can be fortified with nutrients or whether supplementation is already needed.

The results of our work clearly indicated that the standard education of patients with CD should be expanded so that their food choices improve the nutritional value of the diet. However, implementation may be difficult in many countries because of limited access to a registered dietitian with experience in the dietary treatment of CD. Bebb et al. found that only 38% of patients with CD diagnosed on average 5.4 years earlier remained under the care of a specialist [36]. Herman et al. also indicated that patients with CD are not consistently followed up [37]. They found that one and five years after diagnosis, only 3.3% and 15.8%, respectively, had met a registered dietitian. For comparison, only 24% of our patients with the disease lasting 4.8 ± 7.0 years had the opportunity to consult a dietician before the beginning of the study. On the other hand, we noticed that patients with CD are not always interested in long-term care by a dietician due to the need to come to appointments or because they are ashamed to admit that they do not follow a GFD. Despite the fact that the dietician called and messaged each of our patients several times to arrange a follow-up visit, many did not respond; eventually, 24 patients refused to participate in the follow-up.

It is also worth emphasizing that the previous meeting with a dietitian did not affect the SDE result in our study. This may, of course, be due to the small sample size, but there is no doubt that not every dietitian is equally trained in treating CD. Experts emphasize that a dietitian working with a gastroenterologist should be highly knowledgeable about a GFD [16]. In their opinion, one of the most important elements for increasing adherence to a GFD is regular dietary consultation.

In our study we also demonstrated that the dietary education in CD patients does not always have to be based on in-patient visits. Because of the COVID-19 pandemic, and difficulties in accessing a dietitian, a good solution may be to combine face-to-face consultations with follow-ups by e-mail or by phone [38,39]. The results of the studies of Sainsbury et al., Jeanes et al., and Muhammad et al. confirm our suggestion [40,41,42].

The description of difficulties in accessing a dietitian would not be complete if it were not been mentioned that dietary consultations in countries like Poland are not reimbursed, and gastroenterological walk-in clinics are not obligated to employ a dietitian [43]. To summarize, it is important to emphasize that CD dietary care should not be based on just one consultation focusing on gluten sources. Our conclusion is confirmed by the recently published guidelines of the European Society for the Study of Coeliac Disease [16]. Experts emphasize that dietary education is essential not only to learn how to eliminate gluten from the diet, but also to make the patient aware that a GFD, like any diet, must be properly balanced.

There were several limitations to this study. Firstly, we only recruited patients from one medical center, so the sample was relatively small and consisted mostly of women. However, CD is more common in women, and they are more interested in dietary consultation than men. Secondly, although the dietitian thoroughly explained to the study participants how to prepare the 3-day food diary and checked the patient’s food intake during the face-to-face consultation by using 24 h recall, the diary method is subject to random error due to the respondent not admitting to eating a certain food or forgetting to write the food in the diary. Thirdly, concomitant autoimmune diseases could result in avoidance or increased consumption of certain foods.

## 5. Conclusions

In conclusion, we observed that as many as 38% of CD patients did not adhere to a GFD; however, standard dietary consultation by the dietitian experienced in CD treatment, successfully improved this based primarily on the ability to identify gluten sources. Unfortunately, a better GFD compliance did not change the nutritional value of the GFD, except for a lower sodium intake. We observed that patients with CD ate less plant protein (both active CD and in CD remission), but more total fat and energy (especially those in CD remission) than the control group, which may increase the risk of cardiovascular disease or obesity. Moreover, CD subjects did not consume enough calcium, iron, vitamin D, folic acid or fiber, but they ate too much cholesterol. Therefore, the role of a dietitian in the treatment of CD needs to be increased, so that patients not only learn to follow a diet, but also how to balance it.

## Figures and Tables

**Table 1:** The characteristics of the study groups.

Characteristic	All CD Patients (*n* = 72)	Follow-Up CD Group (*n* = 48)	Controls (*n* = 30)
Female (%)	87.5	91.7	90
Age (years)	40.3 ± 11.9	42.3 ± 11.2	36.6 ± 12.1
BMI (kg/m^2^)	22.2 ± 3.6	21.8 ± 3.3	23.1 ± 3.7
Classic presentation of CD (%)	21	15	-
Disease’s duration (years)	4.8 ± 7.0	5.9 ± 8.1	-
Diagnosis over a year ago (%)	68	73	-
Meeting a dietitian before the study (%)	24	29	-
Active CD (%)	36.1	37.5	-
Other autoimmune diseases (%)	36.1	27.1	-

There were no significant differences between the groups. Abbreviations: BMI––body mass index; CD––celiac disease.

**Table 2:** Intake of macronutrients per person, per day in all CD patients (*n* = 72) and controls (*n* = 30).

Observed Component	Recommended	All CD Patients (*n* = 72)		Controls (*n* = 30)	
		Mean ± SD	% of E	Mean ± SD	% of E
Protein (g)	10–20% of E	84 ± 29	16	78 ± 20	18
Plant protein (g)	NA	19 ± 9	-	23 ± 9 *	-
Animal protein (g)	NA	53 ± 26	-	49 ± 21	-
Fat (g)	20–35% of E	93 ± 47	38	75 ± 27 *	37
SFA (g)	as low as possible	31 ± 16	13	29 ± 10	14
MUFA (g)	NA	33 ± 23	13	28 ± 12	13
PUFA (g)	NA	12 ± 9	5	11 ± 5	6
Cholesterol (mg)	NA	324 ± 182	-	333 ± 135	-
Carbohydrates (g)	45–60% of E	247 ± 87	46	222 ± 61	46
Sucrose (g)	NA	56 ± 31	-	45 ± 23	-
Fiber (g)	25 g	23 ± 9	-	22 ± 9	-

* Significant differences (*p* <0.05) between celiac and control subjects. Abbreviations: CD––celiac disease; E––energy; MUFA––monounsaturated fatty acids; NA––not available; PUFA––polyunsaturated fatty acids; SFA––saturated fatty acids.

**Table 3:** Intake of energy and micronutrients per person per day in all CD patients (*n* = 72) and controls (*n* = 30).

Observed Component	All CD Patients (*n* = 72)		Controls (*n* = 30)	
	Mean ± SD	% of DRI	Mean ± SD	% of DRI
Energy (kcal)	2138 ± 718	107	1822 ± 451 *	103
Sodium (mg)	2296 ± 1337	161	2317 ± 825	155
Potassium (mg)	3529 ± 1139	101	3255 ± 947	93
Calcium (mg)	929 ± 470	91	834 ± 237	80
Phosphorus (mg)	1221 ± 454	174	1240 ± 325	177
Magnesium (mg)	344 ± 109	105	306 ± 96	94
Iron (mg)	12 ± 5	81	12 ± 4	82
Zinc (mg)	9 ± 4	107	9 ± 3	113
Vitamin A (µg)	1429 ± 1285	198	960 ± 566	135
Vitamin D (µg)	5 ± 6	31	6 ± 10	40
Vitamin E (mg)	11 ± 8	129	12 ± 6	144
Vitamin B_1_ (mg)	1.2 ± 0.6	105	1.1 ± 0.4	101
Vitamin B_2_ (mg)	1.6 ± 0.6	141	1.5 ± 0.4	130
Niacin (mg)	20 ± 11	141	19 ± 7	133
Vitamin B_6_ (mg)	2.2 ± 0.9	163	1.9 ± 0.6	145
Vitamin B_12_ (µg)	4.7 ± 5	197	4.4 ± 3.2	182
Vitamin C (mg)	135 ± 106	177	116 ± 85	153
Folate (µg)	287 ± 136	72	318 ± 118	80

* Significant differences (*p* < 0.05) between celiac and control subjects. Abbreviations: CD––celiac disease; DRI––dietary recommended intake.

**Table 4:** Intake of macronutrients per person per day in follow-up CD group (*n* = 48) before and after one year of follow-up.

Nutrients	Recommended Values	At Baseline (*n* = 48)		After Follow-Up (*n* = 48)	
		Mean ± SD	% of E	Mean ± SD	% of E
Protein (g)	10–20% of E	81 ± 25	16	78 ± 25	17
Plant protein (g)	NA	18 ± 8	-	17 ± 10	-
Animal protein (g)	NA	51 ± 24	-	49 ± 19	-
Fat (g)	20–35% of E	90 ± 42	39	84 ± 40	37
SFA (g)	as low as possible	31 ± 15	13	30 ± 15	13
MUFA (g)	NA	32 ± 20	13	30 ± 17	13
PUFA (g)	NA	11 ± 7	5	11 ± 7	5
Cholesterol (mg)	NA	297 ± 180	-	299 ± 166	-
Carbohydrates (g)	45–60% of E	235 ± 76	46	238 ± 78	46
Sucrose (g)	NA	50 ± 28	-	51 ± 26	-
Fiber (g)	25 g	22 ± 6	-	22 ± 11	-

There were no significant differences between baseline and after follow-up. Abbreviations: E––Energy; MUFA––monounsaturated fatty acids; NA—not available; PUFA––polyunsaturated fatty acids; SF––saturated fatty acids.

**Table 5:** Intake of energy and micronutrients per person per day in follow-up CD group (*n* = 48) before and after one year of follow-up.

Nutrients	At Baseline (*n* = 48)		After Follow-Up (*n* = 48)	
	Mean ± SD	% of DRI	Mean ± SD	% of DRI
Energy (kcal)	2047 ± 584	104	1972 ± 649	101
Sodium (mg)	2274 ± 1216	159	2011 ± 1060 *	142
Potassium (mg)	3415 ± 941	98	3508 ± 1188	100
Calcium (mg)	842 ± 390	82	800 ± 313	78
Phosphorus (mg)	1147 ± 385	164	1196 ± 366	171
Magnesium (mg)	344 ± 106	105	353 ± 129	108
Iron (mg)	11 ± 4	76	11 ± 4	74
Zinc (mg)	9 ± 4	104	9 ± 3	104
Vitamin A (µg)	1408 ± 1360	199	1069 ± 898	151
Vitamin D (µg)	5 ± 7	32	6 ± 9	39
Vitamin E (mg)	10 ± 6	119	10 ± 7	121
Vitamin B_1_ (mg)	1.2 ± 0.6	105	1.1 ± 0.5	98
Vitamin B_2_ (mg)	1.5 ± 0.6	135	1.5 ± 0.5	132
Niacin (mg)	20 ± 10	142	19 ± 9	135
Vitamin B_6_ (mg)	2.1 ± 0.8	157	2.0 ± 0.9	176
Vitamin B_12_ (µg)	5.1 ± 6.0	211	4.3 ± 4.3	180
Vitamin C (mg)	125 ± 103	168	124 ± 88	163
Folate (µg)	264 ± 87	66	276 ± 107	69

* Significant differences (*p* <0.05) between baseline and after follow-up. Abbreviations: DRI—dietary recommended intake.

## Data Availability

The data presented in this study are available on request from the corresponding author. The data are not publicly available due to the fact that they contain information that could compromise the privacy of research participants.
